# 2-D chemical structure image-based in silico model to predict agonist activity for androgen receptor

**DOI:** 10.1186/s12859-020-03588-1

**Published:** 2020-10-26

**Authors:** Myeong-Sang Yu, Jingyu Lee, Yongmin Lee, Dokyun Na

**Affiliations:** School of Integrative Engineering, Department of Biomedical Engineering, Chung-Ang University, Seoul, Republic of Korea 06974

**Keywords:** Chemical compound images, Convolutional neural network, Androgen receptor toxicity

## Abstract

**Background:**

Abnormal activation of human nuclear hormone receptors disrupts endocrine systems and thereby affects human health. There have been machine learning-based models to predict androgen receptor agonist activity. However, the models were constructed based on limited numerical features such as molecular descriptors and fingerprints.

**Result:**

In this study, instead of the numerical features, 2-D chemical structure images of compounds were used to build an androgen receptor toxicity prediction model. The images may provide unknown features that were not represented by conventional numerical features. As a result, the new strategy resulted in a construction of highly accurate prediction model: Mathews correlation coefficient (MCC) of 0.688, positive predictive value (PPV) of 0.933, sensitivity of 0.519, specificity of 0.998, and overall accuracy of 0.981 in 10-fold cross-validation. Validation on a test dataset showed MCC of 0.370, sensitivity of 0.211, specificity of 0.991, PPV of 0.882, and overall accuracy of 0.801. Our chemical image-based prediction model outperforms conventional models based on numerical features.

**Conclusion:**

Our constructed prediction model successfully classified molecular images into androgen receptor agonists or inactive compounds. The result indicates that 2-D molecular mimetic diagram would be used as another feature to construct molecular activity prediction models.

## Background

Androgen receptor (AR) is one of nuclear receptors playing an important role in expressing male phenotype. AR is activated by steroid hormones such as testosterone and 5α-DHT [1]. Although AR-induced cellular functions are vital for early development and physiological regulations [2], excessive AR activation triggered by xenobiotic agonists accelerates diseases severity such as androgen insensitivity syndrome (AIS) and prostate cancer [3]. For this reason, AR is one of targets for testing drug toxicity, and drug candidates should be assayed for potential AR-mediated toxicity. There have been reports on experimental AR affinity assays of chemical compounds [4, 5]. Recently due to the advance of high-throughput techniques AR screening could be carried out at a large scale [6]. Nonetheless, experiment-based screening methods are still costly and time-consuming as well as it is not possible to cover the structural diversity of chemical compounds. To tackle down the limitation, computational AR-dependent toxicity prediction methods have been developed to save time and cost. However, their accuracies are not enough to completely replace experiments and thus they need to be improved further.

In 2018, a combination of three computational algorithms to predict agonist and antagonist activity on AR and thyroid hormone receptor was published [7]. Although the model predicted nuclear receptor agonist molecules with moderate performance, the model was not accurate enough to substitute experimental screening methods. To our knowledge, though there are several reports on docking-based AR agonist prediction [8, 9], there are no other machine-learning-based in silico approaches to predict AR agonist activity, which can be virtually carried out at high-throughput.

Generally, in silico approaches to predict biological activity of chemical compounds firstly converts a molecular structure into thousands of different molecular features [10]. Various molecular features have been introduced including static features such as physicochemical properties, and dynamic features such as molecular fingerprints. Various conversion methods have been developed for accurate feature generation [11]. Since the molecular features do not represent all the chemical and physical properties of chemical compounds, such conversion necessarily accompanies information loss. Thus, developing a novel conversion method and combining the method with conventional ones could enhance the performance of in silico models by minimizing information loss.

Convolutional neural network (CNN) is a class of deep neural network (DNN) algorithm mainly introduced for image classification [12]. CNN models can effectively extract and learn local features from images with fewer parameters compared with conventional DNN models, by employing multiple convolution and pooling layers [13]. With such advantages, CNN model has been employed to solve various problems including medical image classification [14] and facial expression recognition [15].

In this study, instead of the limited conventional molecular features, we employed the 2-D structure mimetic diagram of chemical compounds (ball-and-stick models) to construct a prediction model. CNN algorithm was introduced to classify molecular images into AR agonists or inactive compounds. We expected CNN model analyzed substructure of input molecules by itself by automatically extracting and learning features from input images. As a result, the constructed CNN-based in silico model successfully classified molecular images to AR agonists or inactive compound, which outperformed previous models in terms of overall accuracy.

## Materials and methods

### Construction of training dataset

**Fig. 1:**
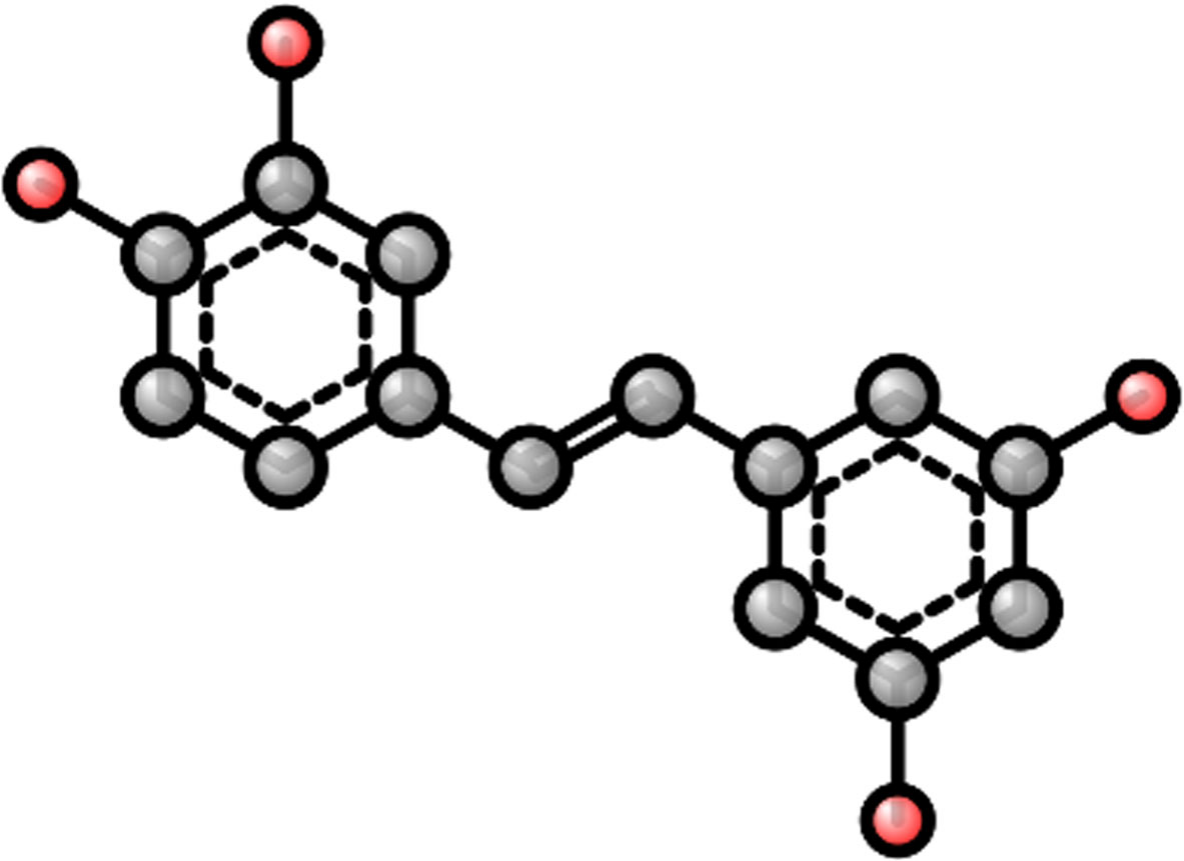
A 2-D image of a chemical compound (piceatannol)

In Tox21 Data Challenge 2014, training dataset for AR-induced toxicity prediction was provided (PubChem AID 743040). In this study, we downloaded the dataset from Tox21 Data Challenge 2014 repository. It contains Simplified Molecular-Input Line-Entry System (SMILES) [16], NCATS Chemical Genomics Center (NCGC) ID and agonist activity (active or inactive) of 9362 compounds. After removing duplicate compounds, we obtained 270 active agonists and 7198 inactive chemicals for AR. We used OpenBabel toolbox (version 2.4.0) [17] to convert compounds from SMILES format to 2-D ball-and-stick structure (Fig. 1). All structures were saved into PNG format, and further transformed into 200 (width) × 200 (height) × 3 (color channels) array with RGB values of each pixel.

### Model construction

**Fig. 2:**
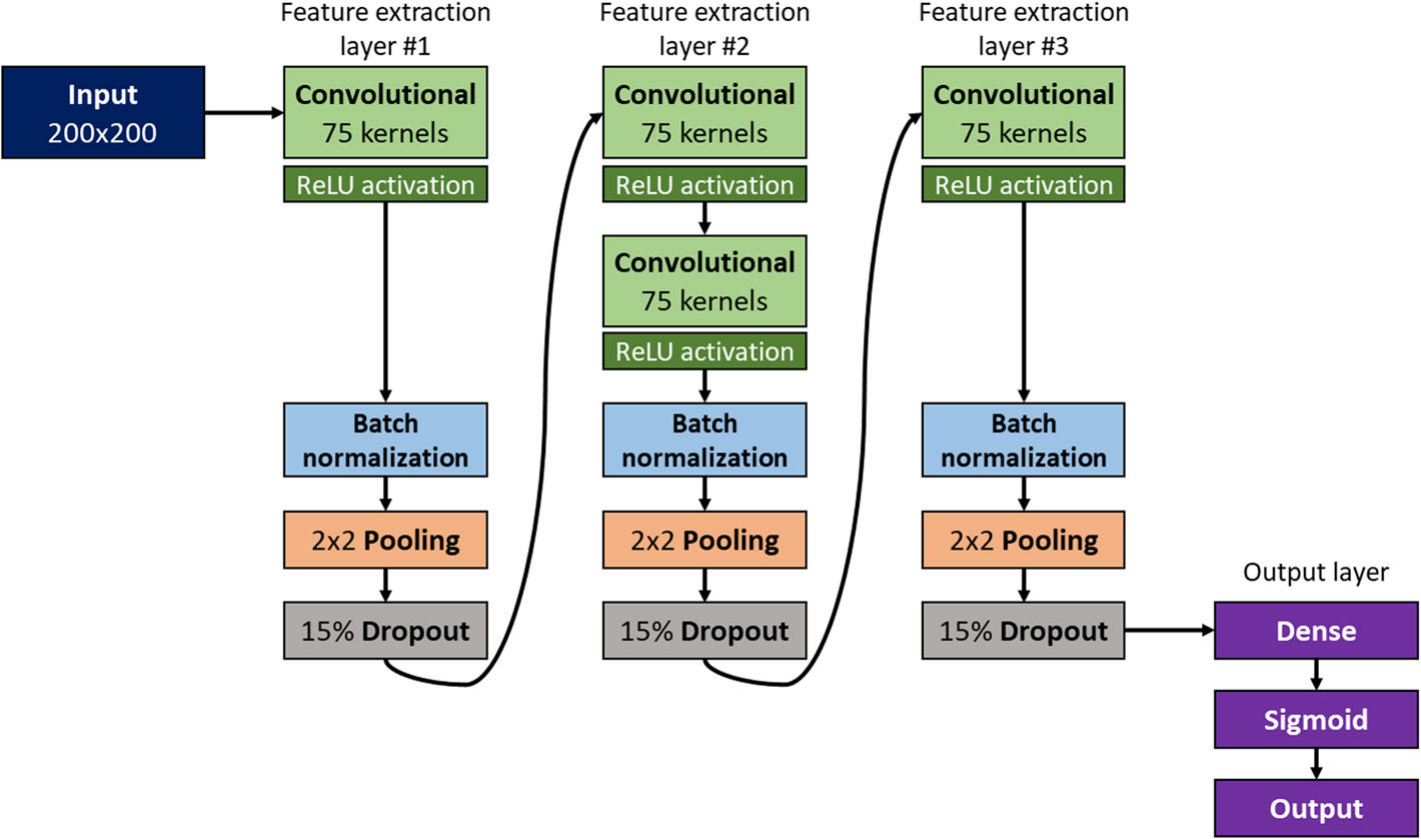
Constructed CNN model architecture

Convolutional neural network (CNN) algorithm was employed to construct a molecular image classification model for AR agonist screening. We constructed a CNN model with a feature extraction part and 1 fully connected output layer. Overall model architecture is shown in Fig. 2.

Feature extraction part consists of convolutional, dropout, pooling and batch normalization layers. Convolutional layers automatically search and extract representative features from input images with convolutional filters and activation function. Dropout, pooling and batch normalization layers were employed to prevent overfitting and reduce computational resource usage by reducing the number of features. After extraction part, representative features will be input features of dense neural network with single layer. As a result, inputted molecular image will be classified into AR agonist or inactive compound.

To enhance the overall performance of prediction model, we optimized two factors: learning rate and L2 regularization factor. Learning rate is a scalar value that determines training speed of the model and controls the rate of adaptation to changing input data. L2 regularization factor suppresses model from overfitting on training data. We evaluated with four learning rates (10^− 3^, 10^− 4^, 10^− 5^, 10^− 6^) and five L2 regularization factors (0.4, 0.6, 0.8, 1.0, 1.2), and constructed 20 different trained models.

The optimal parameters were determined by AUC, and then the threshold to classify AR-toxicity was further optimized by Matthews Correlation Coefficient (MCC) since the training and evaluation datasets were highly unbalanced [18]. However, instead of selecting the highest AUC, we selected a trained model with high AUC and robust prediction accuracies. A trained model may not display robustness in prediction, so we traced AUC results epoch-by-epoch and selected a model that showed stable prediction accuracies and a high AUC.

### Model validation

For model evaluation, we collected active and inactive AR agonists from the literature [7]. The dataset contained agonists, antagonists and inactive compounds for AR. A test dataset was constructed with 71 active AR agonists and 220 inactive compounds, excluding duplicated molecules and compounds included in training dataset. Prediction performance was calculated as MCC, AUC, sensitivity, specificity, accuracy, and positive predictive value (PPV).

We tested whether our model was able to predict AR agonists with high performance, which were collected from other bioassay results. Twenty-five compounds extracted from AR agonist bioassay (AID 639154) were used as a test dataset [19]. The dataset consisted of two active AR agonists and 23 inactive compounds, separated by a threshold of IC50 = 10 μ M.

## Results and discussion

### Training performance

To select the optimal hyperparameters of a CNN model, we constructed 20 models with different parameters. Prediction performances of the trained models in 10-fold cross-validation was recorded epoch by epoch. The parameters and resulting AUC values are shown in Table 1. From the result, the CNN model with learning rate of 10^− 3^ and L2 regularization factor of 1.0 showed the best AUC value (0.915).

**Table 1:** Performance (AUC) results of 20 different CNN models

Learning Rate	L2 regularization factor	AUC	Epoch^a^
10^−3^	0.4	0.881	200
	0.6	0.908	50
	0.8	0.861	195
	1.0	0.915	85
	1.2	0.848	51
10^−4^	0.4	0.893	10
	0.6	0.880	70
	0.8	0.909	31
	1.0	0.905	32
	1.2	0.895	137
10^−5^	0.4	0.861	76
	0.6	0.899	84
	0.8	0.887	68
	1.0	0.881	75
	1.2	0.894	46
10^−6^	0.4	0.878	500
	0.6	0.871	494
	0.8	0.876	463
	1.0	0.902	429
	1.2	0.867	469

^a^Epoch number at which the highest AUC was obtained

**Fig. 3:**
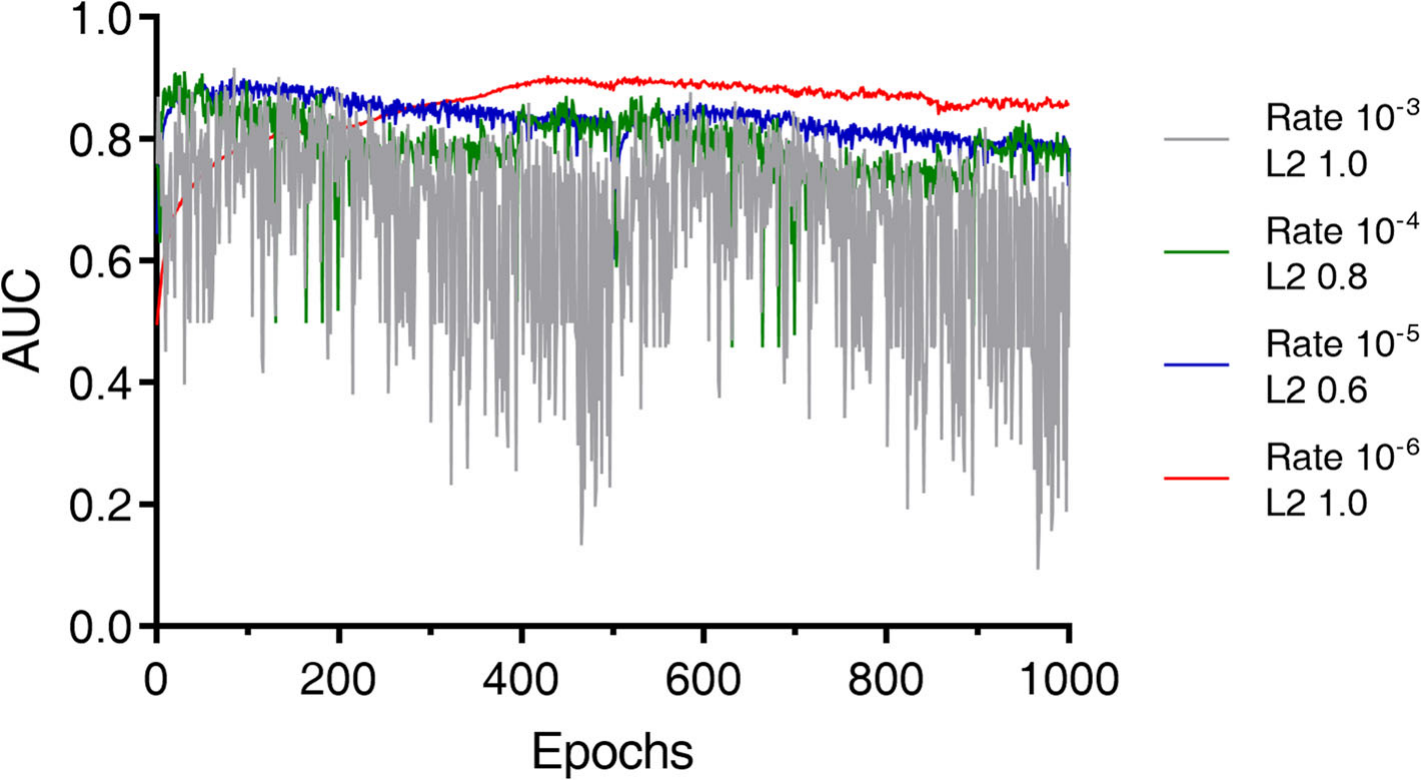
Epoch-by-epoch performance (AUC) results of 4 representative models

The top four models were also analyzed in terms of robustness. As shown in the Fig. 3, under certain parameters the trained models showed unstable performances (fluctuation in AUC value), which represents that the models were overshoot by fast learning rates. Consequently, a learning rate of 10^− 6^, a regularization factor of 1.0, and epoch number of 429 were determined, which showed high AUC (0.902) and stable performance as well.

For further optimization, the optimal threshold value to classify active and inactive agonists was determined in terms of MCC, which is an appropriate index to show performance of classification on imbalanced datasets. The selected best model marked the highest MCC of 0.688 at the threshold of 0.66 was selected, and other classification performances under the threshold are listed in Table 2.

**Table 2:** Performance results under optimal threshold

MCC	Sensitivity	Specificity	PPV	Accuracy
0.688	0.519	0.998	0.933	0.981

### Model test

For the model test, we collected 71 active AR agonists and 220 inactive compounds from the literature [7]. Overall performance values are listed in Table 3. The test results also showed comparable performance with cross-validation result. Interestingly, PPV on the test dataset increased up to 0.882, which represents that once a compound is predicted to be an agonist, then it would be an agonist with high probability.

**Table 3:** Prediction performance on test dataset

AUC	MCC	Sensitivity	Specificity	PPV	Accuracy
0.783	0.370	0.211	0.991	0.882	0.800

**Fig. 4:**
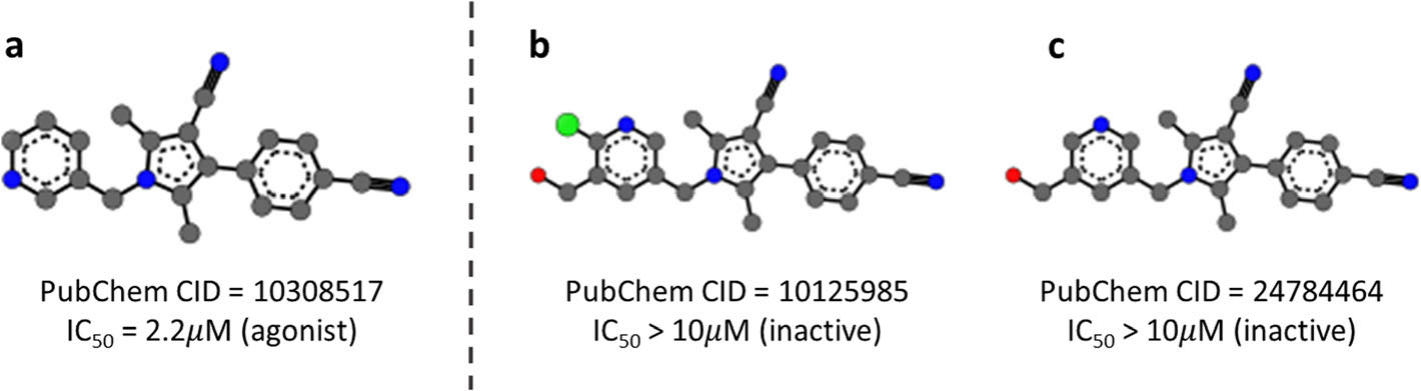
Three representative compounds with same backbone structure collected from external dataset. Compound **a** is AR agonist, while **b** and **c** are inactive compounds

As another evaluation, we used AR agonist activity screening bioassay record as a test dataset. Yamamoto S et al. designed and synthesized a series of 4-phenylpyrrole derivatives from known AR antagonists to discover novel orally available AR antagonists as effective prostate cancer drugs. Antagonist and agonist activity of synthesized compounds were biologically evaluated and reported as a bioassay record (PubChem AID 639154) [19]. As shown in Fig. 4, most of compounds in the dataset are derived from 1-arylmethyl-4-phenylpyrrole and have almost same 2-D diagram. Although such similarity makes AR agonist detection difficult, our constructed model successfully classified all compound exactly, showing 100% accuracy. These results proved that our model can be used to predict AR agonist activity with high accuracy and molecular images can be another feature for predicting biological activities of chemical compounds.

## Conclusion

We introduced a CNN-based model to predict molecular agonist activity for AR with a novel input data: 2-D chemical structure of molecules. Generally, CNN based image classification models can solve real-life problems: handwriting recognition, object recognition, and so on. We also employed the same strategy for image-based AR toxicity classification. Our model marked high performance (AUC = 0.902) in cross-validation and AUC of 0.783 on test dataset, outperforming the previous model (AUC = 0.756) based on classical classification algorithms and classical numerical features in 2018 [7].

We expect our approach can be utilized to predict various biological activities of chemical compounds, e.g. toxicity classifications, absorption classifications, etc. Our model proved that images could be another feature for classification.

## Data Availability

The datasets used and/or analyzed during the current study are available from the corresponding author on reasonable request.

## Abbreviations

AR: Androgen receptor
AUC: Area under ROC curve
CNN: Convolutional neural network
MCC: Matthews correlation coefficient
PPV: Positive predictive value
SEN: Sensitivity
SMILES: Simplified molecular-input line-entry system
SPE: Specificity
